# The ProteomeXchange consortium in 2026: making proteomics data FAIR

**DOI:** 10.1093/nar/gkaf1146

**Published:** 2025-11-06

**Authors:** Eric W Deutsch, Nuno Bandeira, Yasset Perez-Riverol, Vagisha Sharma, Jeremy J Carver, Luis Mendoza, Deepti J Kundu, Chakradhar Bandla, Selvakumar Kamatchinathan, Suresh Hewapathirana, Zhi Sun, Shin Kawano, Shujiro Okuda, Brian Connolly, Brendan MacLean, Michael J MacCoss, Tao Chen, Yunping Zhu, Yasushi Ishihama, Juan Antonio Vizcaíno

**Affiliations:** Institute for Systems Biology, Seattle WA 98109, United States; Center for Computational Mass Spectrometry, University of California, San Diego (UCSD), La Jolla, CA 92093, United States; Department of Computer Science and Engineering, University of California, San Diego (UCSD), La Jolla, CA 92093, United States; Skaggs School of Pharmacy and Pharmaceutical Sciences, University of California, San Diego (UCSD), La Jolla, CA 92093, United States; European Molecular Biology Laboratory, European Bioinformatics Institute (EMBL-EBI), Wellcome Trust Genome Campus, Hinxton, Cambridge, CB10 1SD, United Kingdom; Department of Genome Sciences, University of Washington, Seattle WA 98195, United States; Center for Computational Mass Spectrometry, University of California, San Diego (UCSD), La Jolla, CA 92093, United States; Department of Computer Science and Engineering, University of California, San Diego (UCSD), La Jolla, CA 92093, United States; Skaggs School of Pharmacy and Pharmaceutical Sciences, University of California, San Diego (UCSD), La Jolla, CA 92093, United States; Institute for Systems Biology, Seattle WA 98109, United States; European Molecular Biology Laboratory, European Bioinformatics Institute (EMBL-EBI), Wellcome Trust Genome Campus, Hinxton, Cambridge, CB10 1SD, United Kingdom; European Molecular Biology Laboratory, European Bioinformatics Institute (EMBL-EBI), Wellcome Trust Genome Campus, Hinxton, Cambridge, CB10 1SD, United Kingdom; European Molecular Biology Laboratory, European Bioinformatics Institute (EMBL-EBI), Wellcome Trust Genome Campus, Hinxton, Cambridge, CB10 1SD, United Kingdom; European Molecular Biology Laboratory, European Bioinformatics Institute (EMBL-EBI), Wellcome Trust Genome Campus, Hinxton, Cambridge, CB10 1SD, United Kingdom; Institute for Systems Biology, Seattle WA 98109, United States; School of Frontier Engineering, Kitasato University, Kanagawa 252-0373, Japan; Database Center for Life Science, Joint Support-Center for Data Science Research, Research Organization of Information and Systems, Chiba 277-0871, Japan; Niigata University Graduate School of Medical and Dental Sciences, Niigata 951-8510, Japan; Department of Genome Sciences, University of Washington, Seattle WA 98195, United States; Department of Genome Sciences, University of Washington, Seattle WA 98195, United States; Department of Genome Sciences, University of Washington, Seattle WA 98195, United States; Beijing Proteome Research Center, National Center for Protein Sciences, Beijing Institute of Lifeomics, Beijing 102206, China; Beijing Proteome Research Center, National Center for Protein Sciences, Beijing Institute of Lifeomics, Beijing 102206, China; Graduate School of Pharmaceutical Sciences, Kyoto University, Kyoto 606-8501, Japan; European Molecular Biology Laboratory, European Bioinformatics Institute (EMBL-EBI), Wellcome Trust Genome Campus, Hinxton, Cambridge, CB10 1SD, United Kingdom

## Abstract

The ProteomeXchange consortium of proteomics resources (http://www.proteomexchange.org) was established to standardize open data practices in the mass spectrometry (MS)-based proteomics field. Here, we describe the main developments in ProteomeXchange in the last 3 years. The six member databases of ProteomeXchange, spread out in three different continents, are the PRIDE database, PeptideAtlas, MassIVE, jPOST, iProX, and Panorama Public. We provide updated data submission statistics, showcasing that the number of datasets submitted to ProteomeXchange resources has continued to accelerate every year. Through June 2025, 64 330 datasets had been submitted to ProteomeXchange resources, and from those, 30 097 (47%) just in the last 3 years. We also report on the improvements in the support for the standards developed by the Proteomics Standards Initiative, e.g. for Universal Spectrum Identifiers and for SDRF (Sample and Data Relationship Format)-Proteomics. Additionally, we highlight the increase in data reuse activities of public datasets, including targeted reanalyses of datasets of different proteomics data types, and the development of novel machine learning approaches. Finally, we summarize our plans for the near future, covering the development of resources for controlled-access human proteomics data, and for the support of non-MS proteomics approaches.

## Graphical Abstract

**Graphical Abstract:**
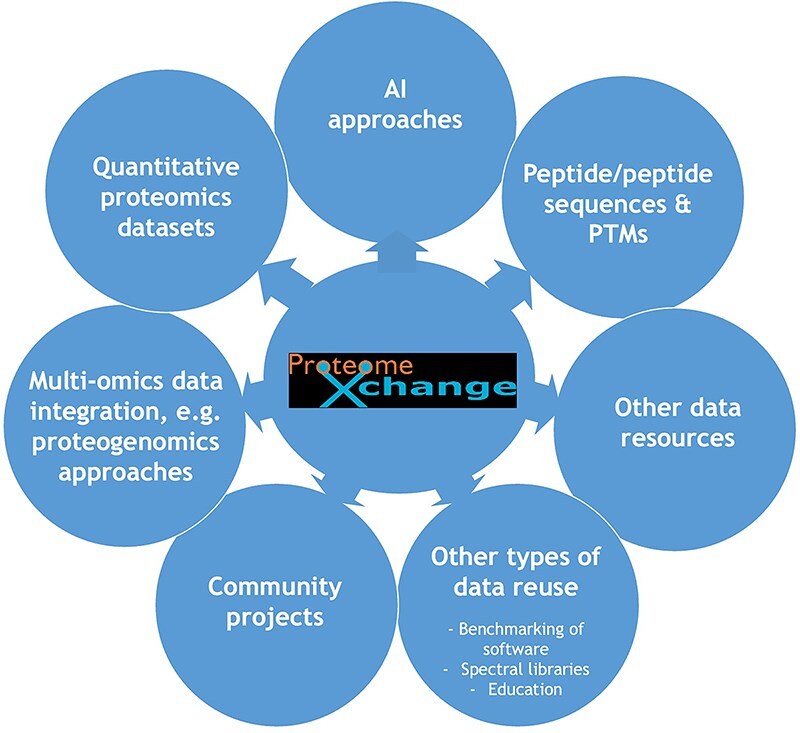

## Introduction

Public data sharing has become the default practice in the proteomics field, which is increasingly prominent in the life sciences as proteomics-only approaches or as part of multi-omics studies. Open data practices have generally been adopted due to the requirements of funding agencies and scientific journals, but also due to the perceived reliability of proteomics public data repositories. In the last decade, there has been a dramatic increase in the amount of proteomics data in the public domain. This has triggered multiple types of data re-use activities that are increasingly contributing to the rapid development of the field, for instance in the context of machine learning (ML) and artificial intelligence (AI) approaches [1], among many others.

Since 2012, the main proteomics data repositories are working together under the umbrella of the ProteomeXchange Consortium (http://www.proteomexchange.org) [2–5]. ProteomeXchange has standardized open data practices internationally via data submission and dissemination of public mass spectrometry (MS)-based proteomics datasets. There are currently six resources that are members of ProteomeXchange: the PRIDE database [6] (European Bioinformatics Institute, EMBL-EBI, Hinxton, UK), MassIVE (University of California San Diego, USA, in 2014) [7], jPOST [8] (Kyoto University and other institutions, Japan), iProX [9] (National Center for Protein Sciences, Beijing, China), and the smaller resources Panorama Public [10] (University of Washington, Seattle, USA), and PASSEL [11] [as part of PeptideAtlas (Institute for Systems Biology, Seattle, USA)]. ProteomeCentral (https://proteomecentral.proteomexchange.org/) is the common web portal to access publicly available datasets across all six ProteomeXchange resources.

ProteomeXchange data resources are committed to implementing the FAIR (Findable, Accessible, Interoperable, Re-usable) principles [12] for biological data, supporting reproducible research. For this reason, ProteomeXchange resources are closely aligned with the activities of the Proteomics Standards Initiative (PSI; https://psidev.info/), the organization that develops community-based open data standards in the field [13, 14]. As a result, ProteomeXchange resources support the main MS-related PSI open data formats, and other PSI standards such as Universal Spectrum Identifiers (USIs) [15], the ProForma 2.0 notation [16], and the PSI-MS controlled vocabulary [17]. This support involves the development and maintenance of several open-source parser libraries to support these standards.

In December 2022, the ProteomeXchange resources were included in the initial list of Global Core Biodata Resources (https://globalbiodata.org/what-we-do/global-core-biodata-resources/) created by the Global Biodata Coalition, recognizing ProteomeXchange as an essential biological resource for the scientific community. The PRIDE database is also a core data resource of ELIXIR (http://www.elixir-europe.org) [18], recognizing its key role in the life sciences ecosystem in Europe.

Here we provide an update of the activities of the ProteomeXchange consortium and its individual resources since the previous update paper was published in *Nucleic Acids Research* 3 years ago [5]. We also describe updated submission statistics, demonstrating the year-on-year increase in the number of proteomics datasets in ProteomeXchange resources. As a key point, we also highlight key ongoing data re-use activities, both in the context of the ProteomeXchange resources and led by third parties, and discuss future developments. For more detailed information about the individual data resources in ProteomeXchange, please see the recent manuscripts in the NAR database issue for each [6, 8, 9].

## Results

### ProteomeXchange current infrastructure

There have not been any substantial changes in the ProteomeXchange data workflow for datasets in the last 3 years. PRIDE, MassIVE, jPOST, and iProX are *universal* archival resources, storing all types of MS-based proteomics experiments, while PASSEL and Panorama Public are *focussed* on targeted proteomics approaches. Table 1 summarizes the main functionality offered by the different ProteomeXchange resources. ProteomeXchange dataset (PXD) identifiers are persistent and unique, and are used as the main dataset identifier for all originally submitted datasets. RPXD identifiers are issued in some cases for reanalysed datasets (i.e. original datasets reanalysed by one of the ProteomeXchange resources). Additionally, MassIVE, jPOST, and iProX use their own identifiers for datasets in addition to the common PXD identifiers for datasets that comply with the ProteomeXchange requirements. In terms of data licenses, most resources assign as default the Creative Commons CC0 license. Panorama Public uses as its default the CC-BY license, which requires attribution, with a CC0 license also available to data submitters.

**Table 1.** Main functionality offered by the ProteomeXchange resources

Functionality	PRIDE	PASSEL	MassIVE	jPOST	iProX	Panorama public	Peptide atlas
**Types of data access**							
Web interface	Yes	Yes	Yes	Yes	Yes	Yes	Yes
Application Programming Interface	Yes	Yes	Yes	Yes	Yes	Yes	Yes
Protocol for file transfer (download/upload)	FTP, Aspera, Globus	FTP	FTP	FTP, HTTPS, PRESTO, TripleStore	HTTPS, Aspera	WebDAV, HTTPS	FTP
Reviewer private access	File download	File download	File download, web interface	File download	File download, web interface	File download, web interface	N/A
							
**General functionality/web visualization**							
Dataset centric view	Yes	Yes	Yes	Yes	Yes	Yes	Yes
Protein centric view across resource	No	Yes	Yes	No	Yes	Yes	Yes
Annotated mass spectra	Yes	Yes	Yes	Yes	Yes	Yes	Yes
USIs	Yes	Yes	Yes	Yes	Yes	No	Yes
Chromatograms	No	Yes	Yes	No	No	Yes	No
							
**Data license**	CC0	CC0	CC0	CC0	CC0	CC-BY (default), CC0 (optional)	CC0

Once a dataset is submitted, all ProteomeXchange resources provide private password-controlled access for reviewers and journal editors during the manuscript review process (private datasets remain unreleased to the public during that process). Once the manuscript is published, the corresponding dataset(s) are made publicly available in each resource and their metadata are also made available via the ProteomeCentral web portal, so that they may easily be found, accessed, and reused. For all submitted datasets, a set of common experimental metadata at the level of each dataset must be sent to ProteomeCentral (the common data model is encoded in the PX-XML format, http://proteomecentral.proteomexchange.org/schemas/proteomeXchange-1.4.0.xsd), together with the raw MS run files and the processed results (identification and/or quantification data).

In the context of data submissions, the FTP file transfer protocol continues to be supported by most of the resources, while Aspera, HTTPS, and WebDAV are also supported in some cases (Table 1). The jPOST repository employs the in-house developed PRESTO upload protocol [19]. In 2024, PRIDE added support for the Globus file transfer service (https://www.globus.org/data-transfer), which is recommended for very large datasets, especially for institutions where it is not possible to use Aspera due to IT restrictions [6] (https://www.ebi.ac.uk/pride/markdownpage/globus).

#### Updates in ProteomeCentral

The ProteomeCentral user interface (https://proteomecentral.proteomexchange.org/) has undergone a complete rewrite to support the ever-increasing number of datasets available in ProteomeXchange. The back-end is based on the PROXI web service interface for datasets (https://github.com/HUPO-PSI/proxi-schemas). It provides advanced filtering, free text search, faceted search, summarization, and pagination via an OpenAPI endpoint. The back end is coupled with a custom JavaScript front end that enables a smooth user experience in searching for datasets of relevance, downloading lists of datasets, summarizing subsets of PXDs, and examining individual datasets. The OpenAPI endpoint is publicly accessible and can be used by other applications. A similar system based on the same tools is being developed for spectral libraries.

#### ProteomeXchange and the PSI: new developments and supported formats

ProteomeXchange resources support the main open PSI data standards for MS: mzML [20] (for MS data), mzIdentML [21], and mzTab [22] (for the representation of peptide/protein identifications and quantification results). SDRF-Proteomics (Sample and Data Relationship Format)-Proteomics is the data standard for encoding sample metadata and experimental design information and their relationship to raw data files [23]. Adoption of SDRF is growing in the community, and therefore, the number of submitted datasets containing this information is also increasing in parallel. Although the most usual way to create SDRF-Proteomics files is by using spreadsheet-based software, the lesSDRF [24] web tool has been developed to facilitate the process. Additionally, some popular analysis tools in the community, such as MaxQuant, are starting to support it as well [25]. Apart from promoting the creation of SDRF-Proteomics files, there are ongoing developments for the extension of the format to support various different proteomics subdomains (e.g. metaproteomics).

In this context, as a unique initiative, the *Journal of Proteome Data and Methods* (JPDM) is a proteomics data journal that accepts data description papers using SDRF spreadsheets as supplementary material. The journal operates in conjunction with jPOST [8]. The jPOST team contacts data submitters, sends them draft SDRF files created based on jPOST data and published papers, and encourages them to review the content and submit back to JPDM. SDRF-Proteomics files are prepared by researchers registering data in the repository, enriching the dataset metadata available. Furthermore, when original data is reused, the data article is cited in addition to the PXD identifier, providing authors with an incentive to justify the extra effort of better describing the dataset metadata.

Recently, the first data descriptor paper using this workflow was published from JPDM [26], featuring supplementary material that includes jeSDRF (JPDM-empowered SDRF) files compliant with SDRF-Proteomics. The jPOST team is extending this approach to datasets from other ProteomeXchange resources, promoting the conversion of metadata for PRIDE datasets created using lesSDRF into the jeSDRF format, and encouraging the original authors to submit it to JPDM.

#### USI services: visualizing every mass spectrum in ProteomeXchange

USI [15] is a standardized method for expressing a multipart key identifier for every spectrum deposited to a ProteomeXchange resource (https://psidev.info/usi), allowing for greater transparency of mass spectral evidence. All ProteomeXchange resources, apart from PanoramaPublic, support them. ProteomeCentral provides an API service and a spectrum visualization tool (https://proteomecentral.proteomexchange.org/usi/) to retrieve any spectrum from any of the ProteomeXchange partners. The central service (ProteomeCentral USI) uses the USI representation to query all resources that implement the PROXI specification and to determine whether and where the spectrum is available. If found, the spectrum is then displayed through the Lorikeet spectrum viewer (http://uwpr.github.io/Lorikeet/).

Every PX partner is also able to display spectra via USIs within their own resource, and their visualization interfaces are referenced from ProteomeCentral. This is a flexible approach because every resource spectrum viewer provides additional functionalities, for example, linking to other datasets, providing additional metadata, or spectrum prediction for the identified peptide in the USI. There have been multiple recent improvements in the support for USIs in the different resources. First, the USI functionality in PRIDE (PRIDE Archive USI, https://www.ebi.ac.uk/pride/archive/usi) can now extract the specified scan directly from the MS raw files via ThermoRawFileParser [27] for Thermo Scientific raw files, providing access to >80% of the raw files in PRIDE. A new spectral viewer has also been recently developed in PRIDE that (i) enables visualization of multiple interpretations for the same spectrum; (ii) compares USI interpretations with predicted spectra; and (iii) provides additional metadata if SDRF is available for the correspondin dataset.

The PeptideAtlas team developed and deployed a new visualization tool and fragment ion annotation algorithm Quetzal [28], enabling more in-depth annotation of Higher-Energy Collisional Dissociation (HCD) spectra, including internal fragments, immonium ions, isotopic label-associated ions, and known contaminant low-mass ions. In addition to returning the spectrum for a given USI, the MassIVE team has also developed USI query tools to find other repository spectra and/or spectral library for the same peptide precursor (https://massive.ucsd.edu/ProteoSAFe/usi.jsp), and has also co-developed the FASST tool for repository-scale searches of spectra of the same or related (e.g. modified) variants of a given USI spectrum [(https://fasst.gnps2.org/fastsearch/, co-developed with the GNPS (Global Natural Products Social) team].

The ProteomeCentral USI system has been updated to include the ability to visualize predicted spectra from MS2PIP [29] alongside the spectra of specified USIs. Spectra are displayed via Lorikeet, but the peak-by-peak annotation display uses the Quetzal annotation engine. A version of Quetzal is also hosted at ProteomeCentral, allowing users to produce publication-quality PDFs and SVGs of annotated spectra via USIs or pasted-in peak lists.

### Data submission statistics

**Figure 1.**
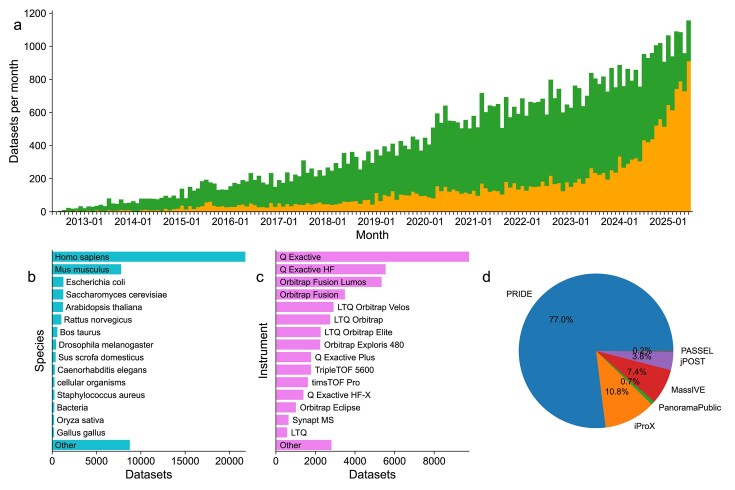
Summary statistics for datasets deposited to ProteomeXchange resources since 2012. (A) Trend in publicly released (green) and not-yet released (orange) datasets from May 2012 through June 2025. A total of 1156 datasets were submitted in June 2025. (B) Summary of the top 15 species for publicly released datasets since 2012. (C) Summary of the top 15 instruments as reported by submitters for publicly released datasets since 2012. (D) Summary of the relative number of all datasets by the receiving repository.

Through the end of June 2025, a total of 64 330 datasets had been submitted to ProteomeXchange resources. Of those, 44 248 datasets (69%) were already publicly available, whereas the rest were still private or unreleased (20 082 datasets, 31%). The number of submitted datasets has increased every year, a trend that has not stopped yet (Fig. 1). In the past 3 years, 30 097 datasets have been submitted to ProteomeXchange resources, meaning that 47% of PXDs were submitted within just the past 36 months through June 2025. This again showcases the continuous significant increase of proteomics datasets in the public domain. During 2024 alone, a record number of 10 686 datasets were submitted to ProteomeXchange resources (890 datasets per month on average). During the first 6 months of 2025, the number of submissions was 6294 datasets (1049 per month on average).

In terms of distribution of datasets submitted across individual ProteomeXchange resources, 49 528 datasets (77%), had been submitted to PRIDE, followed by iProX (6967 datasets, 11%), MassIVE (4770 datasets, 7.4%), jPOST (2443 datasets, 3.8%), Panorama Public (478 datasets, 0.7%), and PeptideAtlas/PASSEL (144 datasets, 0.2%). During the last 3 years, iProX has become the second resource in terms of submitted PXDs. As of September 2025, datasets came from >80 countries, demonstrating further the global reach of ProteomeXchange. The countries with the largest number of submitted datasets were the USA, Germany, China, UK, and France, in this particular order. See Supplementary File 1 for this detailed information, as available for PRIDE.

### Trends in data reuse of public proteomics data

**Figure 2.**
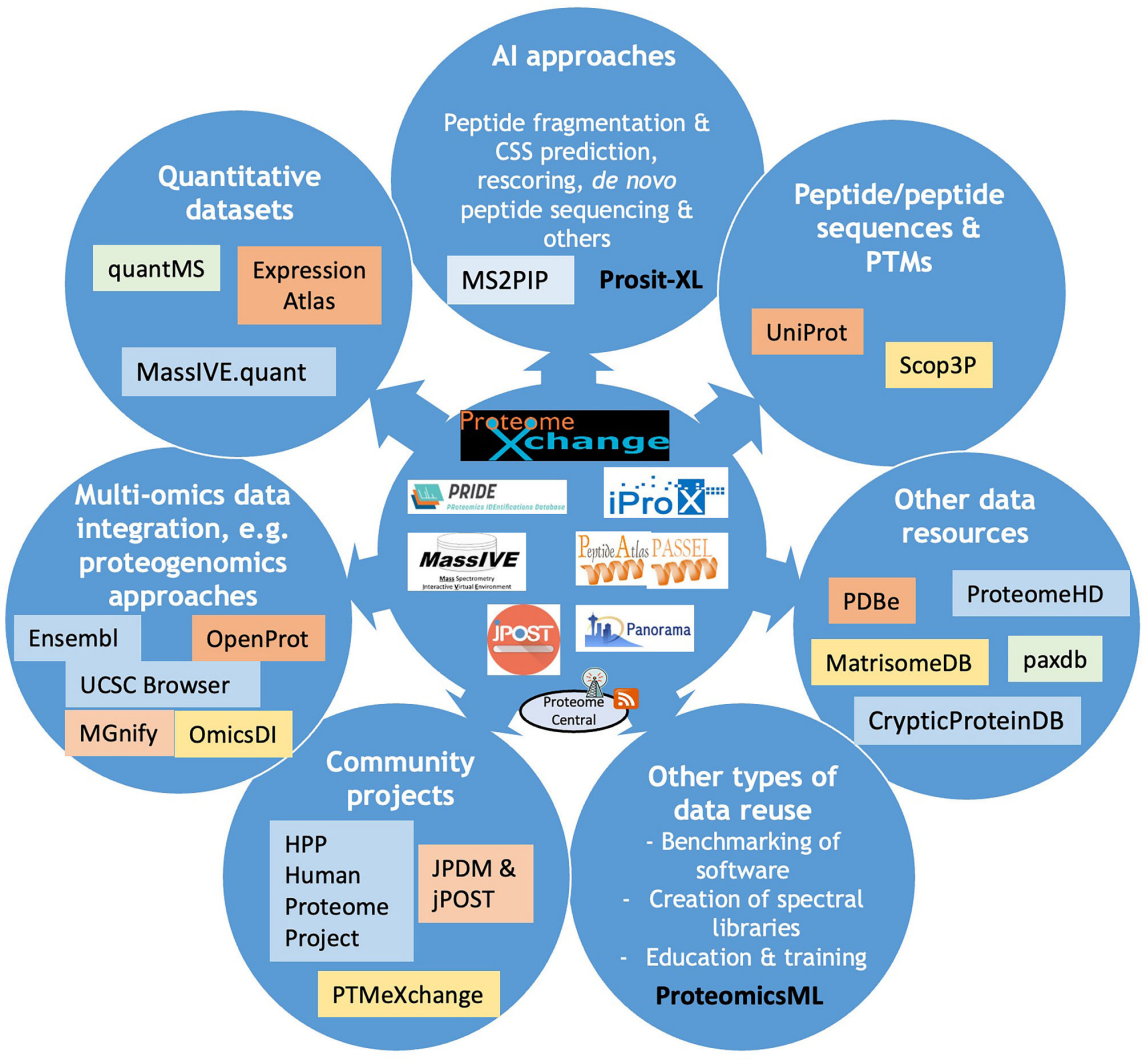
Overview figure including the current ProteomeXchange resources and the main efforts devoted to data reuse of public proteomics datasets. Different types of data reuse are listed and for each of them, the corresponding tools and/or data resources where these data can be accessed are indicated.

As mentioned above, reuse of public proteomics datasets continues to increase and diversify (Fig. 2). In this section, we highlight data reuse activities by the teams behind the ProteomeXchange data resources, involving different data types.

#### Peptide/protein sequence data, including post-translational modifications

ProteomeXchange resources regularly provide peptide and protein sequence data to UniProtKB (UniProt Knowledge Base) [30], the world’s most used protein knowledgebase. In recent years, these efforts have been driven by the Human Proteome Organization Human Proteome Project, aiming to construct the human proteome blueprint [31]. Led by PeptideAtlas, and with the participation of MassIVE, the team has established the protein-level existence of gene products for 93.6% of the human proteome [32], following established community guidelines [33]. This has been the largest community data re-analysis project so far. In addition to the human proteome, PeptideAtlas has continued to create ‘builds’ corresponding to the updated versions of proteomes of various model organisms, reanalysing the relevant public datasets. In recent years, PeptideAtlas has released and analysed the proteomes of *Arabidopsis* [34, 35], maize [36], *Candida albicans* [37], and rice [38].

PTMs are increasingly relevant for explaining protein function and behaviour. However, information about PTMs in databases such as UniProtKB is still limited, and often comes from the literature via manual curation. The PTMeXchange project (https://www.proteomexchange.org/ptmexchange/) was started to improve PTM data availability in UniProtKB, improving its FAIRness. Multiple PTM-enriched datasets from human and several model organisms have been consistently reanalysed, and the resulting high-quality data has been made available in UniProtKB, connecting it to the MS-based proteomics evidence in PeptideAtlas and PRIDE (using USIs). A methodology based on the use of decoy amino acids (mainly alanine) enables the reliable calculation of a false localization rate for phosphorylation [39], a methodology also now extended to other PTMs. The data reanalysis work is organized in ‘builds’ (related groups of datasets), which correspond to the analysis of one particular PTM in one given species. As of August 2025, the builds already finished and integrated in UniProtKB are phosphorylation for rice [40], *Plasmodium falciparum* [41], and the builds covering phosphorylation for other species such as human, mouse, and *Saccharomyces cerevisiae* are about to be integrated in UniProtKB. Additionally, human ‘builds’ from other PTMs are now also at different stages of integration into UniProtKB, including ubiquitination, SUMOylation, lysine acetylation and lysine methylation. In the context of PTM data, it is worth noting that Scop3P [42] is an additional resource that provides access to PTM reanalyses of ProteomeXchange PTM-enriched datasets.

#### Quantitative data reanalysis efforts

The number of reanalysed quantitative proteomics datasets has also increased significantly in recent years. Recognizing the importance of this kind of proteomics data, MassIVE designed and implemented the MassIVE.quant repository infrastructure and data resource [43] for reproducible quantitative MS-based proteomics. Using a branch design, MassIVE.quant stores raw experimental data, metadata of the experimental design, scripts of the quantitative analysis workflow, intermediate input and output files, as well as alternative reanalyses of the same dataset. In this same context, PRIDE has also reanalysed and integrated a wide range of datasets in the resource Expression Atlas [44], where it is possible to access both gene expression and protein abundance data. Most of the integrated datasets come from tissue samples generated in normal/baseline conditions using mainly DDA (Data Dependent Acquisition) but also DIA (Data Independent Acquisition) approaches, including human [45, 46], and model organisms such as mouse, rat [47], and domestic pig [48]. The initial study was performed using mainly cell lines and cancer tissue samples [49]. This reanalysis effort has been followed up by a recent study involving the reanalysis of 12 datasets to detect biomarkers of colorectal cancer [50]. For jPOST, to overcome the differences in highly diverse samples and varying measurement environments and perform quantitative analysis at the repository level, the strategy adopted was to unify data analysis methods for protein identification and to output the protein composition of each individual sample as quantitative values, thereby enabling comparative quantitative analysis between samples. For the former, UniScore was developed as an indicator to integrate and standardize the outputs of multiple search engines in the analysis of DDA data [51]. For the latter, an emPAI algorithm-based spectral count quantification method [52] was adopted, and the reanalysis database is being continually expanded.

##### MassIVE.quant

MassIVE.quant [43] (https://massive.ucsd.edu/ProteoSAFe/static/massive-quant.jsp) is an extension of the MassIVE repository to provide the opportunity for large-scale deposition of data from quantitative MS-based proteomic experiments. MassIVE.quant is compatible with all MS data acquisition types and computational analysis tools. For each dataset, MassIVE.quant systematically stores the raw experimental data, the annotations of the experimental design, the scripts (or descriptions) of every step of the quantitative analysis workflow, and the intermediate input and output files. A branch structure enables MassIVE.quant to store and view alternative reanalyses of the same dataset with various combinations of methods and tools in a way that allows the user to inspect, reproduce or modify any component of the workflow, beginning with well-defined intermediate files. MassIVE.quant also supports infrastructure to fully automate analysis workflows, or to store, and to browse the intermediate results. As of today, MassIVE.quant includes 209 reanalyses of 105 datasets from a variety of species and across all major MS data types.

##### quantms

The quantms project (https://www.quantms.org) is building one of the most comprehensive resources of quantitative proteomics data by systematically reanalyzing public PXDs using an open-source, large-scale quantms pipeline [53]. To date, quantms has processed >103 large-scale human studies (https://quantms.org/datasets), comprising over 29 000 raw MS files from >13 000 samples, and has quantified upwards of 16 000 proteins across tissues, cell lines, and plasma [53]. These reanalyses not only recover more proteins than many of the original studies but also bring consistency and comparability across experiments that were initially produced with very different analytical methods. The resulting harmonized datasets provide a unified view of protein expression at scale, which is invaluable for identifying proteins expressed in specific tissues and conditions. A recent focus of the project is the generation of baseline expression profiles, both in controlled cell models and in clinically relevant samples such as plasma. By integrating iBAQ quantification through the ibaqpy package [54, 55], quantms has created high-resolution expression maps—for example, a corpus of >11 000 proteins quantified across nearly 5800 HeLa MS runs. In plasma, the project has established robust baseline proteomes that can be used to distinguish healthy variability from disease-specific signatures. The quantms workflow continues to be updated by the community (https://github.com/bigbio/quantms). Recent major releases include the support of DIANN 2.1.0, OpenMS 3.4, and major updates on different libraries like pmultiqc and sdrf-pipelines.

##### UniScore-emPAI quantitation

The reanalysis project in jPOST began with developing a methodology to minimize bias in database search engines for protein identification. This involved re-scoring the results from multiple engines on a common scale, merging them, and then controlling the FDR using the target-decoy approach. By defining a very simple UniScore as the sum of the number of product ions matching the sequence with the number of amino acids flanked by two product ions, it became possible to identify more proteins than either individual search engines or combinations of multiple engines [51]. UniScore can be calculated using only product ion information matching sequences, significantly reducing computational resources compared to various existing re-scoring tools. It has already been applied to reanalyse jPOST global proteome and phosphoproteome data, simultaneously providing not only protein identification results but also quantitative data based on the emPAI algorithm [52].

#### Proteogenomics and multiomics data reuse

In this context, proteogenomics data reanalysis efforts should be highlighted first, also including immunopeptidomics and metaproteomics approaches. Public datasets can be reanalysed using sequence databases constructed by e.g. using genomics, transcriptomics or Ribo-seq data, among other DNA/RNA sequencing approaches. The original application of these proteogenomics approaches is to improve genome annotation efforts, by providing proteomics experimental evidence for some genomic events.

It has emerged in recent years that thousands of open reading frames (ORFs) beyond the coding DNA sequences of the core proteome (noncanonical ORFs or ncORFs) seem to undergo some degree of translation and can be detected via MS methods [56], although great care must be taken to avoid false positives when searching for these rare events [57]. Community efforts to assemble sequence databases of these ncORFs based on the highest quality Ribo-seq data are led by GENCODE and the TransCODE consortium [58, 59]. Efforts to document the highest quality evidence with USIs for ncORF detections is led by large-scale reprocessing of human datasets from ProteomeXchange by PeptideAtlas and the TransCODE consortium, finding that detections of ncORFs are quite rare and low abundance in ordinary protease digests without enrichment, but far more commonly detected in immunopeptidome enrichments [56].

Also in the context of noncanonical peptides, we have explored the identification of genome population variants (pangenome) in tissue proteomes [60], investigating the potential impact of pangenomes on future proteomics experiments. In the context of proteogenomics approaches involving microbiome data, a pilot study has been performed involving the integration of metaproteomics data available in PRIDE with the corresponding metagenomics and/or metatranscriptomics data coming from the same samples, in the resource MGnify [61].

All PXDs have been integrated in OmicsDI (Omics Discovery Index) (http://www.omicsdi.org) [62]. This portal facilitates the discoverability of multi-omics public datasets submitted to various public data resources, enabling the link, where possible, between proteomics datasets included in multi-omics studies to the corresponding other types of omics public datasets.

#### Reuse of datasets for AI approaches

Many of the most popular use cases involve the reuse of public datasets for ML or deep learning approaches (e.g. where public datasets are used for training and/or testing purposes), for many different applications, which are revolutionizing the field [63]. The applications include the prediction of peptide fragmentation, prediction of collision cross-section for ion mobility, improvements in algorithms for peptide and protein identification and quantification involving rescoring approaches (e.g. [64–67]), and for the development of *de novo* peptide sequencing algorithms [68], which are being tailored to different data and instrumentation types. For instance, consistently reanalysed public datasets have been used for developing the new peptide fragmentation models of MS2PIP (e.g. for tryptic and nontryptic peptides, and for immunopeptides [29]), Prosit-XL (for crosslinked peptides) [69], and for other peptide types such as glycopeptides [70].

In addition to integrating proteomics data in other resources, one of the main focusses of ProteomeXchange resources will be to continue to provide AI-ready data to the community. In addition to the availability of the mass spectra, this also involves the generation of high-quality reanalysed data (for different data types and approaches), which will need to be well annotated (in terms of experimental metadata), and also provided in suitable data formats.

Recognizing this need, MassIVE has released and continually updates the MassIVE-KB (MassIVE-Knowledge-Base) spectral library [7] constructed from over 1 billion publicly available tandem mass spectra. MassIVE-KB’s latest public release contains reference spectra for 5948 126 peptides and has already enabled the development of multiple cutting-edge AI [71] and other data analysis tools [72]. In addition, the role of ProteomeXchange resources in enabling training and education in AI topics is also key. In that context, we have contributed to the development of the ProteomicsML resource (https://proteomicsml.org/), which provides ready-made datasets for ML models together with tutorials on how to work with them [73].

#### Creation of data resources

Datasets from ProteomeXchange resources are increasingly reused as the basis to create new resources. A few examples are: (i) MatrisomeDB [74], providing an updated view of the human and mouse extracellular matrix; (ii) ProteomeHD [75], a resource providing information about co-expressed proteins; (iii) systeMHC [76], providing a collection of reanalysed immunopeptidomics datasets; (iv) the proteogenomics resource OpenProt [77]; (v) CrypticProteinDB [78], a database of proteome and immunopeptidome derived noncanonical cancer proteins; and (vi) paxDb [79], including protein abundance data from human and several model organisms.

### Additional highlights from the ProteomeXchange member databases

PRIDE has started to use large language models to provide extra functionality. A PRIDE chatbot (https://www.ebi.ac.uk/pride/chatbot/) is available, which has been trained on the PRIDE documentation for external users. The main objective is to help PRIDE users navigate PRIDE documentation, therefore decreasing the time required for the team to reply to ever-increasing support queries. Although the initial implementation was done using open source models [80], as of August 2025, the functionality is provided using the Gemini-1.5-pro model.

iProX has developed a new graphical interface tool iProXplorer to support the data submission of the iProX database (https://www.iprox.cn/page/DownloadClient.html). Specifically, iProXplorer presents a data format to describe experimental metadata and files, supports automated data submission, and exchanges experimental summary between different users. iProXplorer provides a user-friendly interface, as well as a command-line tool that can be integrated into analysis processed. iProXplorer, as an automated submission tool for the iProX database, will help facilitate the development of data sharing in the field of proteomics.

## Discussion and future plans

We have highlighted the main overall developments in ProteomeXchange resources in the last 3 years. The resources will continue to evolve in parallel to the needs of the field. In the context of data archiving activities, PRIDE and MassIVE are currently extending their functionality for supporting controlled-access datasets, such as proteomics datasets generated from human samples collected under study-specific data access restrictions (i.e. datasets that cannot be made openly available), thus requiring repository-supported controlled access capabilities. The need of controlled access options for proteomics data (analogously to genomics/transcriptomics) is mainly due to three reasons: (i) these data could potentially be used to identify research participants; (ii) requirements related to patient consent; and/or (iii) due to personal data regulations like GDPR (General Data Protection Regulation) in Europe, HIPPA (Health Insurance Portability and Accountability Act) in the USA or any other relevant legislation [81].

There is an increasing number of sensitive human proteomics datasets that cannot be made available to the scientific community through a public-access ProteomeXchange resource. We recommend to users at present that if there are any potential legal issues of this type, they should submit their data to an alternative repository outside ProteomeXchange until ProteomeXchange resources can provide controlled access capabilities. However, existing controlled access resources developed for DNA/RNA sequencing data, e.g. the European Genome-Phenome Archive (EGA), the Japanese Genome-Phenome Archive (JGA) and dbGAP, even if they do already store a small number of proteomics datasets at present, are not ideal for proteomics datasets, because their data model cannot appropriately represent such datasets.

There is a growing popularity of non-MS-based proteomics technologies such as the use of affinity reagents (e.g. SomaLogic^®^ and Olink^®^ assays, among others), especially for human plasma datasets. PRIDE is currently starting a new section called PRIDE ‘Affinity Proteomics’ (https://www.ebi.ac.uk/pride/archive/affinity-proteomics) supporting these technologies. If there is a demand for it, the ProteomeXchange framework could be extended in the future to support non-MS data as well. However, it is important to highlight that a very large proportion of the studies coming from non-MS approaches are generated from human samples (and often from cohorts), and then the data may be considered sensitive so that controlled access mechanisms may apply. This is the case, for instance, of the non-MS proteomics datasets generated by UK Biobank, which are available via their dedicated data platform.

In addition, we plan to keep working extensively in data reuse/reanalysis projects, disseminating high-quality proteomics data into other bioinformatics resources, and making AI-ready data to the community. ProteomeXchange remains open to accept new members, provided that they adhere to the consortium requirements set out in the ProteomeXchange Membership Agreement, which was updated in 2024 (https://www.proteomexchange.org/pxcollaborativeagreement_2024.pdf).

## Supplementary Material

gkaf1146_Supplemental_File

## Data Availability

The ProteomeXchange consortium of proteomics resources is freely available to all at http://www.proteomexchange.org.
